# Dynamic mitigation of the tearing mode instability in a collisionless current sheet

**DOI:** 10.1038/s41598-021-91111-8

**Published:** 2021-06-02

**Authors:** Yan-Jun Gu, Shigeo Kawata, Sergei V. Bulanov

**Affiliations:** 1 Institute of Laser Engineering, Osaka University, Suita, Osaka 565-0871 Japan; 2 Institute of Physics of the ASCR, ELI-Beamlines, Na Slovance 2, 18221 Prague, Czech Republic; 3 Graduate School of Engineering & Graduate School of Regional Development and Creativity, Utsunomiya University, Yohtoh 7-1-2, Utsunomiya, 321-8585 Japan; 4 Kansai Photon Science Institute, National Institutes for Quantum and Radiological Science and Technology, 8-1-7 Umemidai, Kizugawa-shi, Kyoto 619-0215 Japan

**Keywords:** Laser-produced plasmas, Magnetically confined plasmas

## Abstract

Dynamic mitigation for the tearing mode instability in the current sheet in collisionless plasmas is demonstrated by applying a wobbling electron current beam. The initial small amplitude modulations imposed on the current sheet induce the electric current filamentation and the reconnection of the magnetic field lines. When the wobbling or oscillatory motion is added from the electron beam having a form of a thin layer moving along the current sheet, the perturbation phase is mixed and consequently the instability growth is saturated remarkably, like in the case of the feed-forward control.

## Introduction

The dynamic mitigation of plasma and fluid instability was proposed in Refs.^1–4^. This approach uses the superimposing the phase-controlled plasma perturbations with the modulations growing due to the instability developing. As a result the instability growth reduces, like in the case of the feed-forward control^5,6^, by an energy-carrying driver introducing perturbations into plasma systems parameters. If the perturbation phase is controlled by, for example, wobbling motion of driver beam, the superimposed overall perturbation amplitude can be saturated.

**Figure 1:**
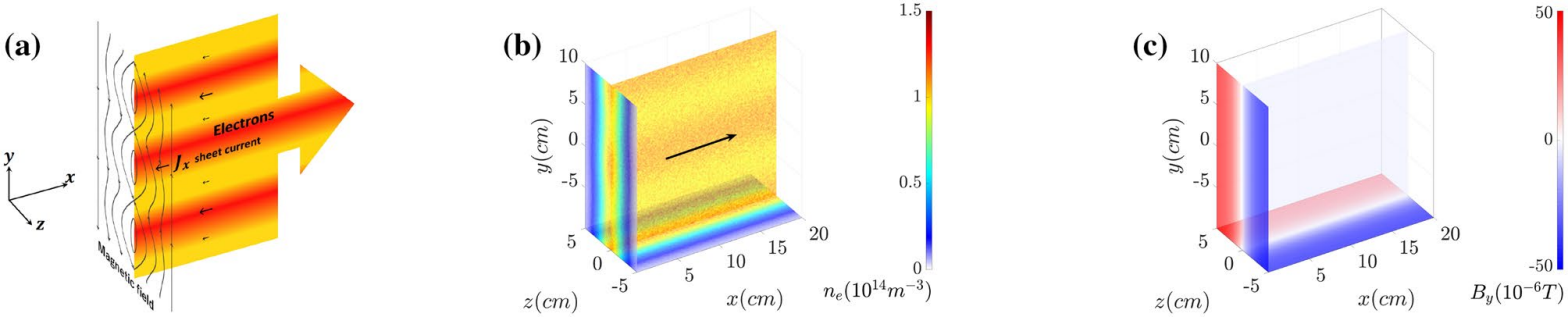
(**a**) Schematic of plasma system sustained by electron current sheet. Magnetic reconnection is induced along with the plasma filamentation. The initial conditions of (**b**) the electron number density $n_e$ and (**c**) the magnetic field are also presented.

Here with the particle-in-cell simulations, we demonstrate the dynamic mitigation of the tearing mode instability of the current sheet in collisionless plasma. Theory and 3-dimensional (3-D) simulations show a clear mitigation of the electron current filamentation growth studied in Refs.^7–11^ corresponding to magnetic reconnection^12–22^. A current sheet in a plasma creates an anti-parallel magnetic field with the magnetic field changing direction in the electron current sheet as shown in Fig. 1a. The growth of the tearing mode instability causes magnetic reconnection. The current sheet formation in high electric conductivity plasmas can be found in various situations: for example, magnetic reconnection via the current sheet formation and disruption is considered as a basic mechanism of solar flares^23^, magnetic reconnection in the current sheets on the day side and in the tail of the earth magnetosphere plays the key role in the high energy charged particle acceleration^24,25^, and magnetic reconnection is crucially important in developing controlled magnetic confinement fusion^26,27^. Magnetic reconnection studies with high power laser interaction with matter is a fast developing research direction in so-called laboratory astrophysics^28–30^.

## Results

The equilibrium state for the current sheet in a collisionless plasma based on the Harris solution presented in Ref.^31^ is used as an initial configuration in the computer simulations whose results are discussed below. In the case under consideration, the current sheet is located in the $z=0$ plane with electric current directed along the *x* axis as shown in Fig. 1b. According to Ref.^31^ the distribution function of the $j=e,i$ particle species is given by

1 $$\begin{aligned} f_j(\mathbf{v},z)=\frac{n_j}{(2\pi v_{Tj}^2)^{3/2} \cosh ^2(z/L)}\exp \left[ -\frac{(v_x-V_j)^2+v_y^2+v_z^2}{2 v_{Tj}^2}\right] , \end{aligned}$$

where $\mathbf{v}=(v_x,v_y,v_z)$ (for describing the initial equilibrium we assume a non-relativistic approximation), $V_j$ and $v_{Tj}$ are the mean velocity and thermal velocity of the *j* species particle, whose temperatures are given by $T_j=m_j v_{Tj}^2/2$. Under the condition

2 $$\begin{aligned} \frac{V_e}{T_e}=-\frac{V_i}{T_i}, \end{aligned}$$

the electron density and ion density are equal to each other $n_e(y)=n_i(y)=n(y)$. For the sake of simlicity we assume that $T_e=T_i=T$, i. e. $V_e=-V_i=V$. In this case, the density and magnetic field dependences on the coordinate *z* are

3 $$\begin{aligned} n(z)=\frac{n}{ \cosh ^2(z/L)} \end{aligned}$$

with the maximum of the density at $z=0$ and

4 $$\begin{aligned} \mathbf{B}(z)=B_0\,{ \tanh (z/L)} \mathbf{e}_y, \end{aligned}$$

with $B_0=(8\pi n T)^{1/2}$, i.e. the magnetic field changes the sign at $z=0$, and $\mathbf{e}_y$ is the unit vector in the *y* direction. The current sheet thickness *L* is equal to

5 $$\begin{aligned} L=\frac{c}{V}\left( \frac{T}{4\pi n e^2}\right) ^{1/2}=\frac{\lambda _D}{\beta } \end{aligned}$$

with $\lambda _D$ and $\beta =V/c$ being the Debye lenght and the electron (ion) average velocity normalized on the speed of light in vacuum, *c*. Further, the particle in cell simulations are carried out in the frame of reference moving along the *x* axis with the normalized velocity equal to $\beta =0.1$. In the boosted frame of reference the ions are at the rest, the electrons move with the velocity $-2 c \beta$, and the electron and ion density are related to each other as $n_e=n_i+2 \beta ^2 n/\gamma$, where $\gamma =1/\sqrt{1-\beta ^2}$. Since the drift velocities of the particles are supposed to be classical in our considerations, the electron density is then approximately $n_e=n_i+2 \beta ^2 n$. The electric field arising from the electric charge separation is expressed by $\mathbf{E}=\gamma \beta B_0 \tanh ( z/L) \mathbf{e}_z$.

The initial small perturbations are imposed on the electron density $n_e$ along the *y* direction. The perturbations are periodic with the amplitude of 5% and the wavelength of $L_y= 10 \; \text{cm}$.

In our simulations we employ 3D particle-in-cell code EPOCH^32^. The simulation box is ${20~\text{cm}\times 20~\text{cm}\times 20~\text{cm}}$, and the maximum electron density is $n_0 = {1.0 \times 10^{8}\; \text{cm}^{-3}}$. The corresponding mesh cells are $200\times 200\times 200$ with 64 particles in each cell. The details of the simulations and the code information are presented in “Methods”.

For the chosen electron temperature, $10\,$eV, and the energy of the electron motion $m_ec^2 \beta ^2/2$, the scale lengths of the density and magnetic field in the current sheet, $\lambda _D/\beta \approx {2.35~\text{cm}}$, and of the perturbation wavelength, $L_y$, in our system are smaller than the electron inertial length $c/\omega _{pe} \approx {53~\text{cm}}$, which is equal to the electron Larmor radius $r_{Be}=m_e v_{Te} c/e B_0$, here $v_{Te}=\sqrt{2T_e/m_e}\approx 0.006c$ is the electron thermal velocity.

**Figure 2:**
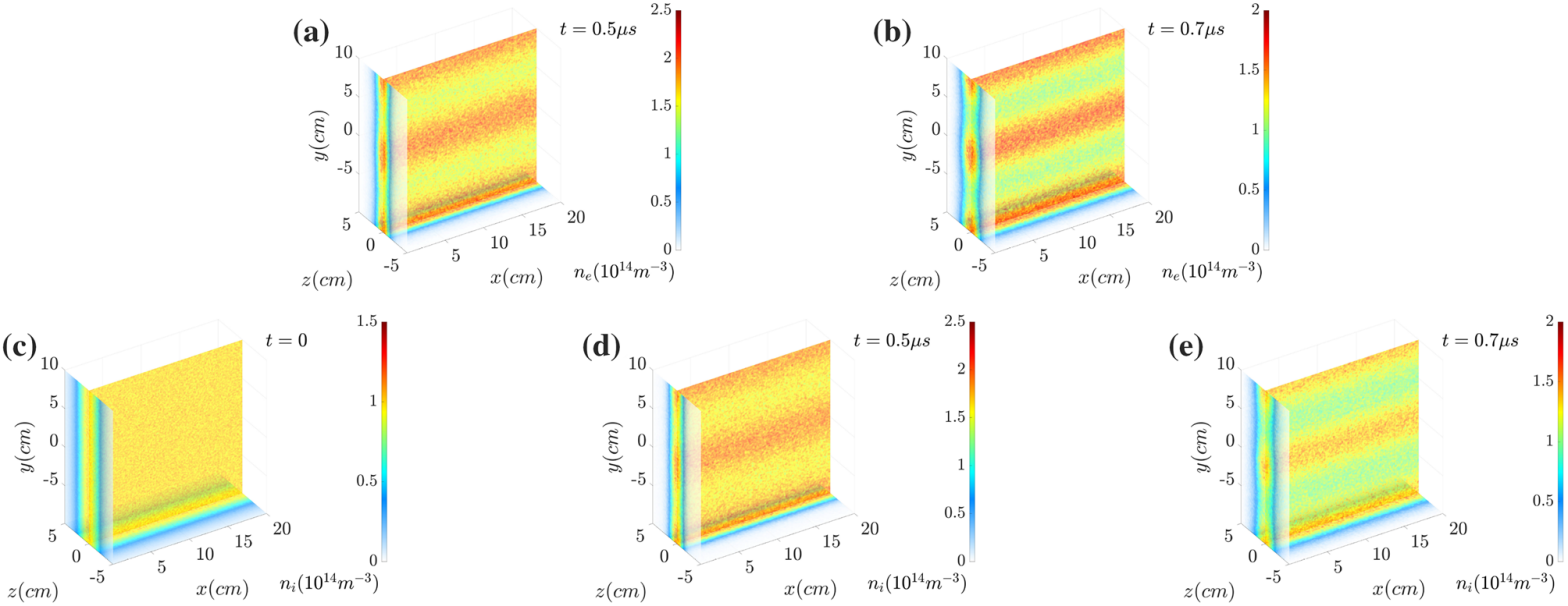
Spatial distribution of the electron number density $n_e$ at (**a**) $t={0.5~\upmu \text{s}}$ and (**b**) $t={0.7~\upmu \text{s}}$, and the proton density $n_i$ at (**c**) $t=0$, (**d**) $t={0.5~\upmu \text{s}}$ and (**e**) $t={0.7~\upmu \text{s}}$. The electron sheet current is filamented along the $z=0$ plane, and the ions also follow the filamentation.

Figure 2 show the spatial distributions of the electron number density $n_e$ at (a) $t= {0.5~\upmu \text{s}}$ and (b) $t= {0.7~\upmu \text{s}}$, and of the proton density $n_i$ at (c) $t=0.0$, (d) $t= {0.5~\upmu \text{s}}$ and (e) $t= {0.7~\upmu \text{s}}$. The electron current sheet breaks up into the filaments along the $z=0$ plane. The ion density also follows the filamentation.

**Figure 3:**
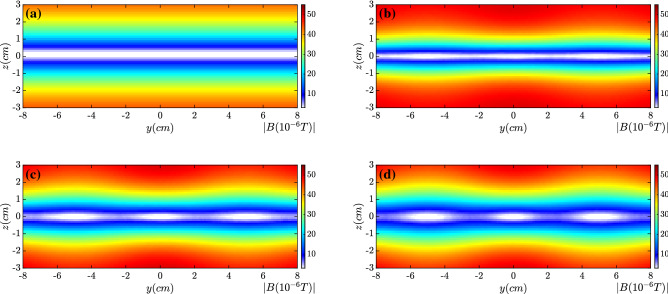
Distribution of magnetic field strength at $x=0$ at (**a**) $t=0.0$, (**b**) $t= {0.5~\upmu \text{s}}$, (**c**) $t= {0.7~\upmu \text{s}}$ and (**d**) $t={0.8~\upmu \text{s}}$. After $t={0.5~\upmu \text{s}}$ magnetic reconnection becomes distinct.

The distributions of magnetic field strength along $x=0$ are presented in Fig. 3 at (a) $t=0.0$, (b) $t={0.5~\upmu \text{s}}$, (c) $t={0.7~\upmu \text{s}}$ and (d) $t= {0.8~\upmu \text{s}}$, respectively. At $z=0$ the initial magnetic field changes the sign. The magnetic island formation and the associated magnetic reconnection become remarkable around time $t= {0.5~\upmu \text{s}}$. The growth rate of the tearing mode instability may be estimated by $\Gamma _{rec}\sim v_A/L$^20^, which is about $\Gamma _{rec}\sim {2.18\times 10^{6}/\text{s}}$ in our cases. The Alfven speed $v_A$ is $v_A\sim {2.18\times 10^6 \; \text{cm}/\text{s}}$. The modulation scale length *L* is approximately equal to 1 cm. The growth time scale can be estimated as $\tau \sim 1/\Gamma _{rec} \sim {0.458 ~\upmu \text{s}}$, which is well consistent with the kinetic simulation results. Around $t={0.8~\upmu \text{s}}$ the tearing mode instability enters the nonlinear stage. Several magnetic islands have formed in Fig. 3d.

**Figure 4:**
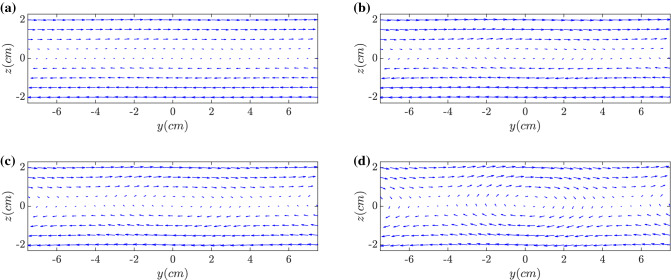
Distributions of magnetic field vectors along $x=0$ at (**a**) $t= {0.2~\upmu \text{s}}$, (**b**) $t= {0.5~\upmu \text{s}}$, (**c**) $t= {0.7~\upmu \text{s}}$ and (**d**) $t= {1.0~\upmu \text{s}}$. After $t= {0.5~\upmu \text{s}}$ magnetic reconnection becomes distinct.

The corresponding magnetic field vector evolutions are shown in Fig. 4 in the plane of $x=0$. Initially, the magnetic vectors are anti-parallel according to $z=0$. With time evolving, the magnetic field lines bend and gradually form the X-point (null-point) with magnetic reconnection, which is clear in Fig. 4d at about ${1~\upmu \text{s}}$. It can be seen that the tearing mode gives rise to the formation of a magnetic island centred in the region of ${-3<\text{y}(\text{cm})<3}$. Magnetic field-lines situated outside are displaced by the tearing mode, but still maintain their original topology. By contrast, field-lines inside the region have been reconnected with a different topology.

**Figure 5:**
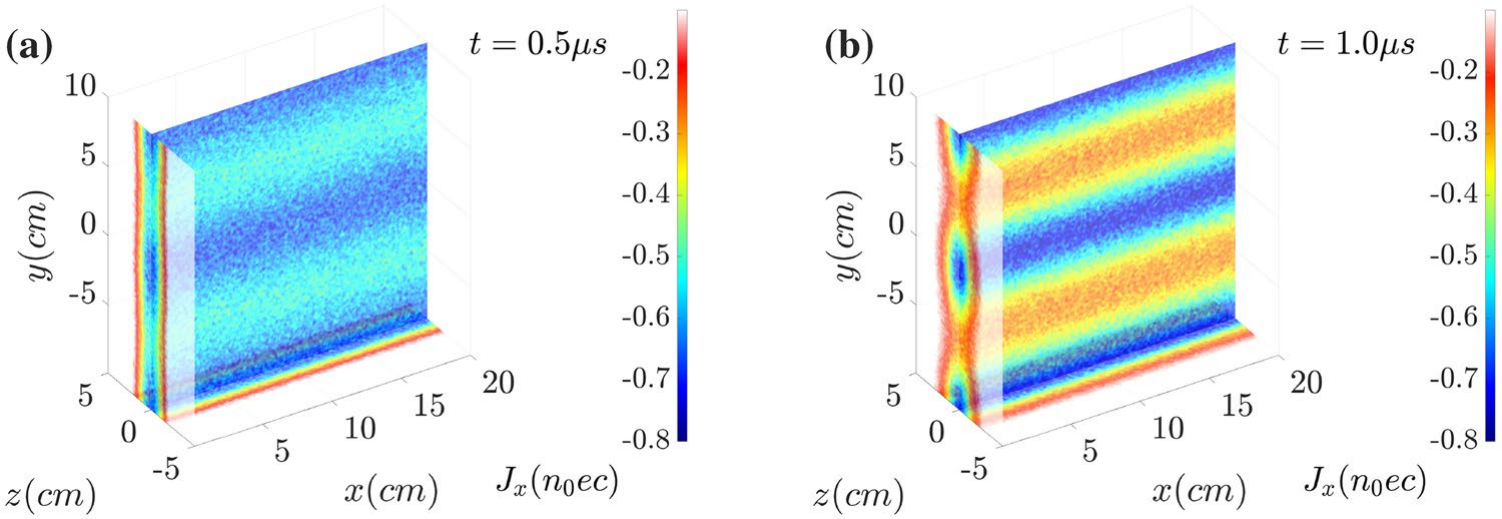
Distributions of current near the electron current sheet $J_x$ at (**a**) $t= {0.5~\upmu \text{s}}$ and (**b**) $t= {1.0~\upmu \text{s}}$. The net current shows the filamentation along with magnetic reconnection.

In Fig. 5, the slices of current density distribution in the plane of $x=0$ (the cross section plane), $z=0$ (the longitudinal plane) and $y= {-10~\text{cm}}$ (the bottom plane) are shown at (a) $t= {0.5~\upmu \text{s}}$ and (b) $t= {1.0~\upmu \text{s}}$. The current density $J_x$ is normalized by $n_0 ec$. The electron current sheet according to the reconnection X-point is formed in Fig. 5a and is significantly amplified in (b). The net current shows the filamentation along with magnetic reconnection.

Now we apply the mechanism of the instability dynamic mitigation to the case of the tearing mode developing in the current sheet. The main purpose of dynamic mitigation mechanism is to saturate the instability growth. It also leads to the smoothing of the modulations in plasmas and fluids, which has been proposed and discussed in detail in Ref.^1–4^. When in an unstable system the perturbations of a physical quantity $\phi$ depend on time and coordinates in the form of

6 $$\begin{aligned} \delta \phi =\delta \phi _0 e^{\Gamma (t-\tau )+i\mathbf{k}\cdot \mathbf{x}+i\Omega \tau }, \end{aligned}$$

the time dependence of modulations $\delta \phi$ is characterized by the growth rate of $\Gamma >0$. Here $\delta \phi _0$ is the perturbation amplitude, $\mathbf{k}$ the wave number vector, $\tau$ the time at which the perturbation is applied, and $\Omega$ defines the perturbation phase. In plasmas, it would be difficult to measure the perturbation phase, and therefore, the feedback control^5^ cannot be directly applied to suppress the plasma instability growth. However, the perturbation phase $\Omega \tau$ would be defined externally by, for example, energy-carrying driver oscillation. When the perturbations introduced at $t=\tau$ change the phase continuously by $\Omega \tau$, the overall perturbation superimposed at *t* is obtained by

7 $$\begin{aligned} \int _{0}^t d\tau \delta \phi _0 e^{i\Omega \tau }e^{\Gamma (t-\tau )+i\mathbf{k}\cdot \mathbf{x}} \propto \frac{\Gamma +i\Omega }{\Gamma ^2+\Omega ^2} \delta \phi _0 e^{\Gamma t} e^{i\mathbf{k}\cdot \mathbf{x}}. \end{aligned}$$

Although the growth rate $\Gamma$ does not change, the perturbation amplitude is well reduced by the factor of $\sim \Gamma /\Omega$ for $\Gamma \ge \Omega$, compared with that non-phase-oscillation case. The theoretical consideration suggests that the frequency $\Omega$ in the perturbation phase change should be larger than or at least comparable to $\Gamma$ for the effective mitigation or smoothing of the perturbations.

**Figure 6:**
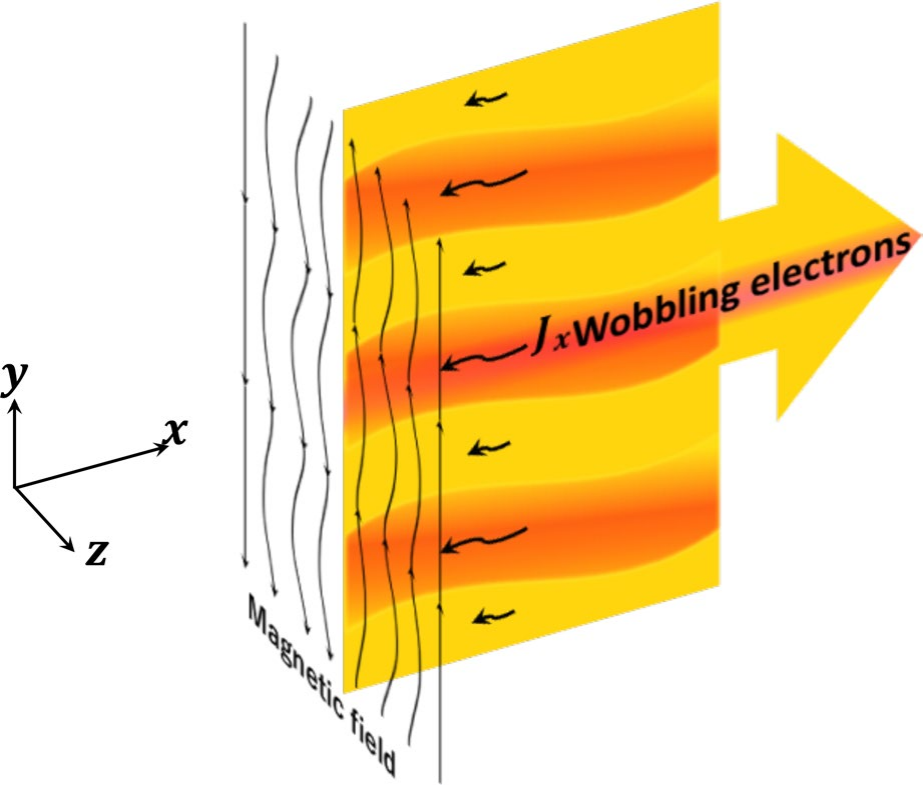
Schematic diagram for dynamic phase control in a plasma system sustained by an electron current sheet, in which electron filamentation and magnetic reconnection grow. The electron sheet current is oscillated along the sheet, and the perturbation phases introduced by the electron beam smooth and mitigate the perturbation amplitude.

Here the phase-control dynamic mitigation mechanism is applied to the case of an electron current sheet plasma as shown in Fig. 1. The electron beam has a form of thin layer periodic along the *y* coordinate with the amplitude of ${10\; \text{cm}}$, the wavelength in *x*-direction of ${10\;\text{cm}}$ and with the wobbling frequency $\Omega _0$ = 300 MHz, which is large enough compared with the reconnection growth rate $\Gamma _{rec}$ : $\Omega _0>> \Gamma _{rec}$. The corresponding schematic of the dynamic phase control in a plasma system sustained by an electron current sheet is presented in Fig. 6. Since the wobbling electron current sheet is modulated periodically along the current sheet, it is expected that the perturbation phases introduced by the electron beam to smooth and mitigate the instability amplitude compared with the non-wobbling case mentioned above.

**Figure 7:**
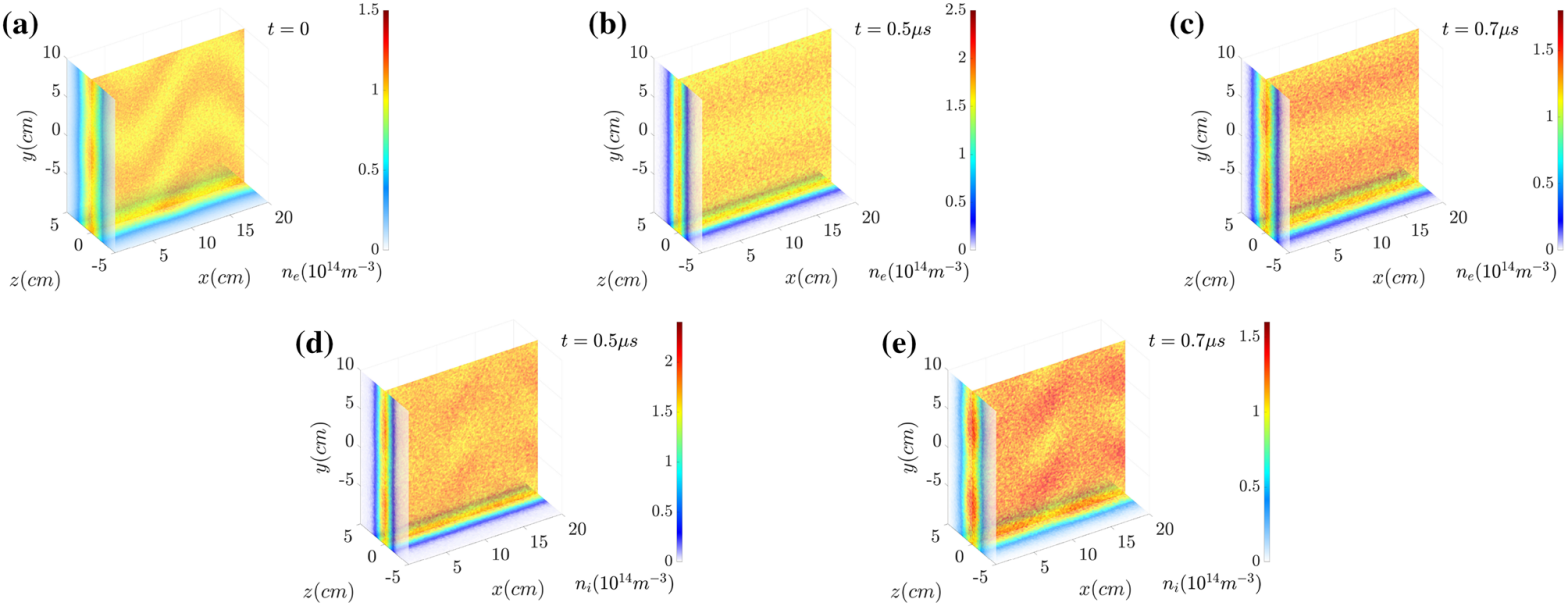
Electron number density $n_e$ at (**a**) $t=0.0$, (**b**) $t= {0.5~\upmu \text{s}}$ and (**c**) $t= {0.7~\upmu \text{s}}$, and the proton density $n_i$ at (**d**) $t= {0.5~\upmu \text{s}}$ and (**e**) $t= {0.7~\upmu \text{s}}$. In this figure the filamentation does not grow well compared with the results in Fig. 2.

**Figure 8:**
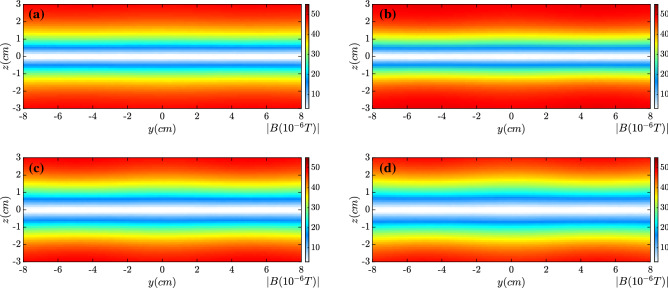
Distribution of magnetic field strength along $x=0$ at (**a**) $t= {0.3~\upmu \text{s}}$, (**b**) $t= {0.5~\upmu \text{s}}$, (**c**) $t= {0.7~\upmu \text{s}}$ and (**d**) $t= {0.8~\upmu \text{s}}$. By the electron wobbling motion along *y*, magnetic reconnection is mitigated well.

Figure 7 show the electron number density $n_e$ and the proton density $n_i$ in the orthogonal slice planes ($x=0$, $z=0$ and $y= {-10\;\text{cm}}$). The initial distribution of the oscillating or wobbling motion of the electron sheet current is presented in Fig. 7a. The effects of dynamic mitigation can be clearly seen by comparing the distributions shown in Fig. 7 with Fig. 2. The filamentations (for both electrons and ions) do not grow significantly. Figure 8 show the magnetic field strength at $x=0$. Due to the suppression of filamentation and the corresponding tearing mode instability, the magnetic field reconnection is also not as remarkable as the non-wobbling case. Comparing with the results presented in Fig. 3, the dynamic mitigation or onset delay of magnetic reconnection is clearly shown in Fig. 8. There is no X-point or tearing induced separatrix and the magnetic fields are still anti-parallel with respect to the boundary along $z=0$.

**Figure 9:**
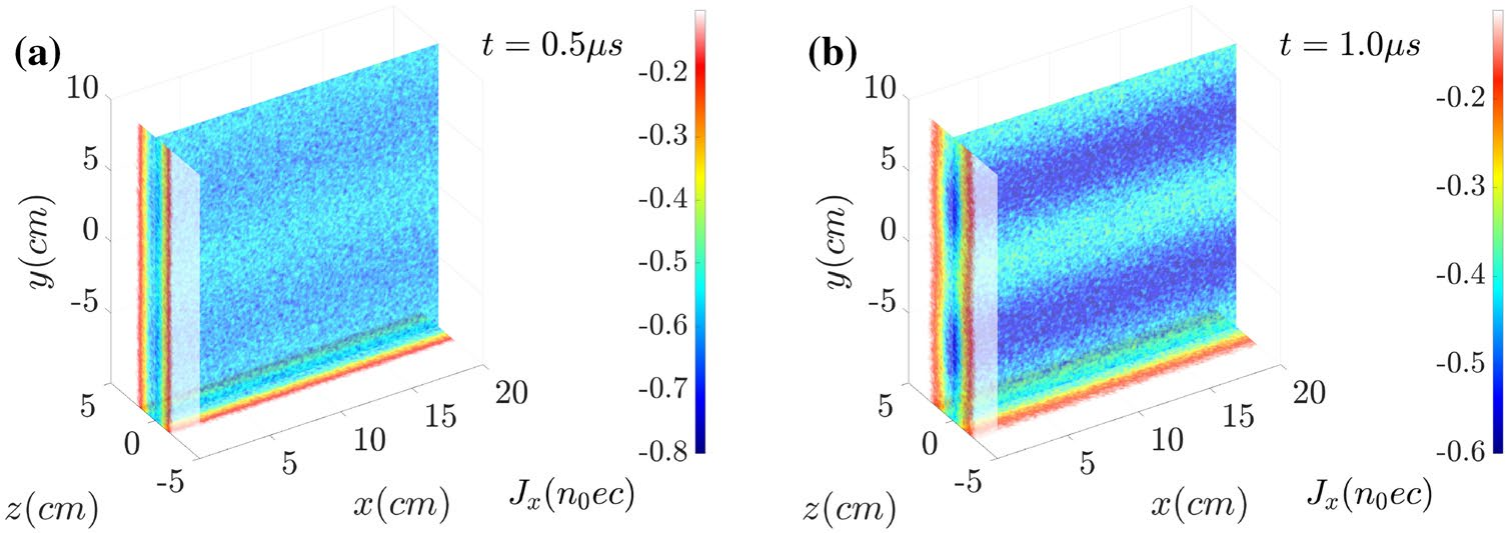
Distributions of current near the electron current sheet $J_x$ at (**a**) $t= {0.5~\upmu \text{s}}$ and (**b**) $t= {1.0~\upmu \text{s}}$. By the wobbling or oscillating motion of the sheet electron current along *y*, the onsets of the filamentation and magnetic reconnection are delayed and mitigated clearly.

In the electric current distributions $J_x$ at (a) $t= {0.5~\upmu \text{s}}$ and (b) $t= {1.0~\upmu \text{s}}$ shown in Fig. 9, although the current density is pinched, the corresponding amplitude becomes much weaker. The current amplitude in the non-wobbling case shown in Fig. 5 ranges from $-0.8~n_0ec$ to $-0.2~n_0ec$. However, experiencing the mitigation, the current amplitude oscillates from $-0.6~n_0ec$ to $-0.4~n_0ec$, which is about 1/3 of the previous amplitude.

**Figure 10:**
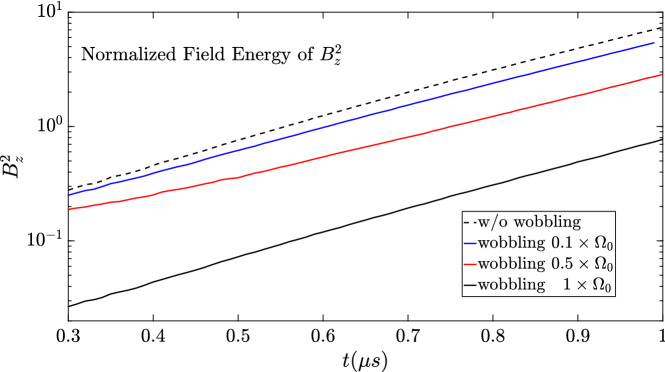
Histories of the normalized field energy of $B_z^2$ for the sheet electron current plasma systems with (solid black line) and without (dotted black line) the wobbling or oscillating motion of the sheet electron current along *y*. The onsets of the filamentation and magnetic reconnection are delayed and mitigated clearly by the dynamic phase control. The solid blue and red curves correspond to the case of wobbling motion with $0.1\Omega _0$ and $0.5\Omega _0$, respectively.

In order to compare the filamentation and magnetic reconnection in the electron current sheet sustained plasma system in the case with and without wobbling mitigation, the histories of the normalized field energy of $B_z^2$ are presented in Fig. 10. In our systems in Figs. 1a and 6, the major energy is the electron kinetic energy. The perturbations imposed initially lead to the electron current filamentation along with magnetic reconnection. Associated with the electron current filamentation and magnetic reconnection, the magnetic field normal component $B_z$ is induced. Figure 10 demonstrates the clear difference in the field energy between the two cases. With the wobbling motion of the electron beam (see Figs. 6 and 7a), the onset of the filamentation and of magnetic reconnection in the current sheet sustained plasma system have been significantly delayed. To prove the robustness of the dynamical mitigation effect, further 3D simulations are launched with the wobbling frequency of $0.1\Omega _0$ and $0.5\Omega _0$. The corresponding evolutions of $B_z^2$ are shown in Fig. 10 as the solid blue and red lines. According to the numerical results, the growth rates of the tearing mode instability, measured by $\sqrt{B_z^2}$, are 2.12 MHz, 2.09 MHz, 2.05 MHz and 2.18 MHz with respect to the cases of non-wobbling, wobbling with $0.1\Omega _0$, $0.5\Omega _0$ and $\Omega _0$. The growth rates obtained numerically are well consistent with the theoretical estimate of $\Gamma _{rec}\sim {2.18~\text{MHz}}$. It presents that the reduction of the perturbation amplitude is proportional to the wobbling frequency while the growth rate of the instability is preserved.

## Discussions and conclusions

The theoretical considerations and 3D numerical computations show the clear effectiveness and viability of the dynamic phase control method to mitigate the tearing instability and magnetic reconnection process. The current sheet plasma system can be found in magnetic fusion devices, space, terrestrial magnetic system, etc. The dynamic mitigation mechanism may contribute to mitigate the magnetic fusion plasma disruptive behavior or to understand the stable structure of the sheet current sustained plasma system. In this paper we focus on the non-relativistic magnetic reconnection and the filamentation (tearing mode like) instability, and the major energy carrier is the sheet electron current. On the other hand relativistic magnetic reconnection has been also studied in space, solar system, planetary magnetic field, etc.^13,33,34^. In those cases, the main energy is carried by the magnetic field and the electromagnetic field. In the relativistic magnetic reconnection case, the dynamic mitigation mechanism should be further studied.

## Methods

The simulations are performed with the 3D version of the relativistic electromagnetic code EPOCH^32^. The simulation box has the size of $L_x \times L_y \times L_z = {20 \;\text{cm} \times 20 \;\text{cm} \times 20 \;\text{cm}}$. The mesh size in the simulations is $\delta x=\delta y=\delta z={0.1 \; \text{cm}}$. 64 quasiparticles per cell are employed with a total number of $5.12\times 10^8$. The real mass ratio of electron and proton ($m_p/m_e=1836$) is used in the simulations. Open boundary conditions for fields and reflection boundary conditions for particles are applied in the *z*-direction. Periodic boundary conditions are employed for both particles and fields in the *x*- and *y*- directions. Both the electrons and protons have the initial temperature of 10 eV with a Maxwellian distribution. The electron beams are introduced on all mesh grids in space simultaneously.

## Data Availability

The data that support the plots and findings of this paper are available from the corresponding author upon reasonable request.
